# Zulu Men’s Conceptions, Understanding, and Experiences of Voluntary Medical Male Circumcision in KwaZulu-Natal, South Africa

**DOI:** 10.1177/1557988319892437

**Published:** 2020-03-05

**Authors:** Celenkosini Thembelenkosini Nxumalo, Gugu Gladness Mchunu

**Affiliations:** 1 KZN Department of Health, Ndwedwe Community Health Centre, Verulam, KwaZulu-Natal, South Africa; 2 Discipline of Nursing, School of Nursing and Public Health, University of KwaZulu-Natal, Durban, KwaZulu-Natal, South Africa

**Keywords:** Masculinity ideology, gender issues and sexual orientation, behavioral research, qualitative research, health awareness, health care issues

## Abstract

Voluntary Medical Male Circumcision (VMMC) is proven to reduce transmission of HIV/AIDS. Despite concerted efforts to scale up VMMC in men aged 18–49, the number of medically circumcised men in this age group remains suboptimal. Research has shown that several individual factors hinder and promote uptake of VMMC. The nature of these factors is not clearly understood within the dimensions of religion, culture and tradition, particularly in a low-income rural setting. This study aimed to analyze Zulu men’s conceptions, understanding and experiences regarding VMMC in KwaZulu-Natal (KZN), South Africa. A qualitative phenomenographic study approach was used to collect data from 20 uncircumcised males at six different clinics that provide VMMC services. Ethical approval to collect data was obtained from the Biomedical Research Ethics Committee of the University of KZN (BREC – BE627/18). Individual in-depth face to face interviews were conducted using a semistructured interview guide. Audiotapes were used to record interviews which were transcribed verbatim and then analyzed manually. The conceptions regarding medical circumcision appeared to be related to religious and cultural beliefs surrounding circumcision and the historical traditional practice thereof. The understanding of males regarding VMMC was mainly attributed to HIV prevention; however, knowledge on the degree of partial protection appeared to be limited. An array of negative accounted in the form of complications such as poor wound healing and postoperative pain undergone by peers and other close influencers’ accounted for participants’ experiences of VMMC. Poor knowledge and negative experiences relating to VMMC could account for reasons why men choose not to undergo VMMC.

Voluntary medical male circumcision (VMMC) was introduced in KwaZulu-Natal (KZN), South Africa, in response to the World Health Organization’s (WHO) recommendations that medical circumcision be adopted by regions of high HIV prevalence (Dickson et al., 2011). Three randomized control trials conducted in South Africa, Kenya, and Uganda proved that VMMC could prevent transmission of HIV by up to 60% in female to male sexual encounters (Auvert et al., 2005; Bailey et al., 2007; Gray et al., 2007). Mathematical modeling on VMMC has reported that scaling up the service to reach 80% of uncircumcised males particularly in the age range of 18–49 could result in a cost saving of at least 16 billion US dollars in HIV infection-related health care expenditure (Hankins et al., 2016).

The review of incidence and prevalence data on HIV infection shows that South Africa, particularly KZN, contributes significantly to the global burden of the pandemic (Wand et al., 2019). Moreover, the highest number of infection-related mortality and morbidity is found in this region. In response to this, the provincial government launched VMMC in KZN with the aim of achieving immediate coverage of sexually active boys and men in the 18–49 year age group (Montague et al., 2014)

Although the initial uptake of VMMC was robust, with just over 1 million medical circumcisions done from 2010 to May 2018, the number of sexually active men circumcised has been below the provincial and national targets (Department of Health, 2018). The number of uncircumcised men undergoing medical circumcision in KZN public health facilities has been suboptimal (Carrasco et al., 2019).

Research done on the uptake of VMMC amongst males has shown that several barriers and drivers hinder and influence the uptake of VMMC by males (Hankins et al., 2016; Nanteza et al., 2018; Rodriguez et al., 2019). Among these are: (1) individuals’ perceptual factors related to fear of pain, procedural complications, and fear of the HIV testing; (2) social factors such as norms, values, and cultural and religious beliefs; (3) social influences such as female partner roles, the media and other public figures, and role models (Abunah et al., 2016; Jones et al., 2018; Nanteza et al., 2018; Osaki et al., 2015; Rodriguez et al., 2019; Wambura et al., 2017).

In Southern Africa, the social norms of religion, culture, and tradition heavily influence men’s decision to undergo VMMC. South Africa in particular is a predominantly Christian nation. The bible states that “In Christ Jesus, neither circumcision nor uncircumcision count for anything” (Galatians 5:6). Certain Charismatic Christian denominations oppose the practice of VMMC because of the above-mentioned scripture. Others believe that Christians should practice circumcision since Jesus was circumcised. In both instances, the choice to undergo VMMC is influenced by an individual’s position in terms of religious belief.

According to Khumalo-Sakutukwa et al. (2013), culture significantly affects individual perceptions and acceptability of circumcision because of the meanings people attach to the procedure. Studies have shown that certain ethnic groups that have not historically practiced ritual circumcision disapprove of circumcision in general (Kitara et al., 2013; Ortblad et al., 2018). In settings where circumcision is practiced for traditional reasons, it was reported that males would disapprove of medical circumcision because of the significance of traditional circumcision in terms of their identity as men (Mark et al., 2012; Rennie et al., 2015).

It can thus be postulated that one of the key elements to the successful scale up of VMMC is understanding the contextual drivers and barriers promoting and hindering the uptake of medical circumcision by male clients. In an attempt to understand these factors several studies have been conducted in sub-Saharan Africa to explore and describe the acceptability of medical circumcision, motivators and obstacles influencing medical circumcision, together with individuals’ perceptions, knowledge, and attitudes regarding the procedure (Jones et al., 2018; Khumalo-Sakutukwa et al., 2013; Lukobo & Bailey, 2007; Wambura et al., 2017; Westercamp & Bailey, 2007). While these have been influential in providing guidance on the type of interventions necessary to scale up VMMC, such interventions have not yet resulted in an uptake that reaches desirable levels.

In addition, research done on the uptake of VMMC in sub-Saharan Africa, including South Africa, has been contextual in design, but has not explicitly alluded to the specific contextual factors and how they actually influence or hinder the uptake of VMMC by males. There has been a casual mention of these factors without an in-depth description of how they unfold and manifest, particularly in a setting where culture and religion heavily influence VMMC.

This study aims to analyze the conceptions, understanding, and experiences of Zulu men regarding VMMC in KZN. This study formed part of a larger study conducted to develop and analyze the qualitative differences in primary health care stakeholders’ experiences, understanding, and conceptions of VMMC in KZN, South Africa. Since the sample comprised uncircumcised Zulu men, the experiences reported in this study are not related to a lived experience of VMMC, but rather to a secondary experience of the phenomenon.

## Research Methodology

The research study used a qualitative phenomenographic design as the intention was to obtain in-depth knowledge on the experiences, understanding, and conceptions of VMMC. Phenomenography, which is different from phenomenology, is a design that aims to discern the qualitatively different ways in which individuals experience, conceptualize, and understand a phenomenon. Phenomenography arises from a study by Ference Martin conducted in 1970 (Stenfors-Hayes et al., 2013), which sought to understand the qualitatively unique ways in which students understood and experienced learning (Limberg, 2008; Martin & Booth, 1997). Based on the initial assumption that students’ experiences and understanding of learning were diverse, the researchers sought to determine the extent of this diversity in terms of quality; thus data collection took on a qualitative approach so that comprehensive accounts of this uniqueness could be clearly demonstrated.

In health sciences, particularly medicine and nursing, phenomenography has been used either explicitly or implicitly to generate knowledge that will influence both clinical practice and education in the fields. According to Sjöström and Dahlgren (2002), the application of a phenomenographic research tradition to nursing research has implications for healthcare service delivery in the sense that it allows for a generation of knowledge on patients’ differing experiences of care; the results thus being that health care professionals take different measures to meet the unique needs of patients. Frank et al. (2009) also state that understanding the differences in patients’ experiences about their illnesses and associated treatment interventions helps in the provision of tailored and holistic individualized care for these patients.

## Research Setting

The study was conducted at six different clinics offering VMMC services within six selected health districts in KZN, South Africa. The clinics are: (1) Sundumbili community health center; (2) Edumbe community health center; (3) Estcourt hospital gateway clinic; (4) Mosvold hospital gateway clinic; (5) Greytown hospital gateway clinic; and (6) Pholela community health center. The number of patients served by these facilities ranges from18,000 to 48,000 per year. All of the above-mentioned facilities offer VMMC services to a target population of 1,000–3,500 men and boys. However, these facilities are only currently able to circumcise around 700–1,500 boys and men per year.

## Research Population

Uncircumcised Zulu men between the ages of 18 and 49 who had no self-reported history of undergoing medical male circumcision in their lifetime. Circumcised men and those not within the above-mentioned age group were excluded from the study as well as those with limited autonomy such as mentally ill patients.

## Sampling

Purposive sampling was used to identify the health districts and clinics where data were collected, and was also used to recruit participants for the study. Inclusion and exclusion criteria were applied when recruiting males to participate in the study. These males were recruited through mediated access while waiting to be attended to in the waiting area of each clinic. A total of 20 uncircumcised Zulu men were recruited to participate in the study. Recruitment ceased as data were saturated at the 20th participant.

## Data Collection

Data collection took place following ethical approval from the Biomedical Research Ethics Committee of the University of KZN (BE 627/18) and the KZN department of health research ethics committee. Research data were collected from males after they had been attended to at the clinic. A self-developed, semistructured interview guide was used to conduct interviews which were recorded and transcribed verbatim. The interview schedule was written in English and translated to IsiZulu (the native language). It consisted of a demographic section and guiding interview questions about participants’ experiences, understanding, and conceptions of VMMC.

Informed consent was obtained from participants first verbally and then in writing before the actual data collection process. Interviews were conducted individually in a private consulting room in the clinics. An audiotape was used to record the interviews which were conducted in IsiZulu and then translated into English, following transcription.

## Data Management

After data collection, the recorded data were stored in a password-protected folder which only the authors and those related to the research had access to. The audio files and interview transcripts are currently stored according to UKZN policy and will be discarded accordingly after a period of 5 years.

## Data Analysis

Data analysis was an iterative process which followed steps guided by Sjöström and Dahlgren (2002) for phenomenographic data analysis. The first step entailed the familiarization, where transcripts were read several times to become familiar with their contents. Second, a more focused reading was done in order to deduce differences and similarities in the transcripts. Through this process, the researcher was able to identify the key elements in the answers. During the condensation step, only the meaningful and relevant aspects of the transcript were extrapolated. The fourth step entailed preliminary grouping in which the focus was on locating and classifying similar responses into preliminary groups. These preliminary groups were reviewed to ascertain whether any other groups showed the same meaning under different headings. This was followed by a preliminary comparison of categories. Thereafter, categories were named following their confirmation in order to emphasize their essence. In the final step, the hope is to discover the outcome space based on their internal relationships and the qualitatively unique ways of understanding, conceiving, and experiencing the phenomena.

## Rigor and Trustworthiness

Lincoln and Guba (1985) proposed four criteria for ensuring the trustworthiness of a qualitative research: (1) credibility was ensured by using an audiotape to record interviews, which, were then transcribed verbatim and analyzed thematically; (2) the assistance of a language specialist was sought during the transcription so that the meaning of the data would not be lost; this ensured dependability; (3) to ensure confirmability, data analysis was conducted in collaboration with an expert in qualitative research methods; and (4) purposively selecting the data collection sites and participants facilitated transferability. The detailed description of study design employed in the study allows for replication of the study in a different setting.

## Ethical Considerations

Ethical approval to conduct the study was obtained from the University of KwaZulu-Natal’s Biomedical Research Ethics Committee (BREC – BE627/18). The provincial and district department of health also provided approval to conduct the study. The clinical sites where data were collected were also asked for permission to conduct the study. Ethical principles of informed consent, confidentiality, privacy, and respect for persons were followed throughout the study

## Research Findings

A total of twenty uncircumcised males from six different health districts participated in the study. The demographic details of these males can be seen in Table 1. The researcher analyzed the data using phenomenographic data analysis processes and identified categories of description relating to uncircumcised males’ experiences, understanding, and conceptions of VMMC in KZN, South Africa. A summary of the major categories of description emerging from this study are presented in Table 2.

**Table1.** Profile of Participants.

Participant	Gender	Participants age	Ethnic group	Regions/cultural belief	Relationship status	Circumcision status	Highest level of education
Participant 1	Male	19	African	New Testament Christian	Single	Uncircumcised	Grade 12
Participant 2	Male	36	African	African traditional religion	Married	Traditionally circumcised	College diploma
Participant 3	Male	42	African	African traditional religion	Married	Uncircumcised	Grade 5
Participant 4	Male	29	African	New Testament Christian	In a relationship	Uncircumcised	University degree
Participant 5	Male	24	African	New Testament Christian	In a relationship	Uncircumcised	Grade 12
Participant 6	Male	27	African	African traditional religion	In a relationship	Uncircumcised	Grade 12
Participant 7	Male	38	African	None	Single	Uncircumcised	College diploma
Participant 8	Male	44	African	Old Testament Christian	Married	Uncircumcised	Grade 8
Participant 9	Male	18	African	New Testament Christian	Single	Uncircumcised	Grade 11
Participant 10	Male	22	African	None	In a relationship	Uncircumcised	Grade 12
Participant 11	Male	31	African	African traditional religion	In a relationship	Uncircumcised	Grade 12
Participant 12	Male	34	African	New Testament Christian	Married	Uncircumcised	College certificate
Participant 13	Male	43	African	New Testament Christian	Married	Uncircumcised	Grade 12
Participant 14	Male	23	African	None	In a relationship	Uncircumcised	College diploma
Participant 15	Male	24	African	New Testament Christian	Single	Uncircumcised	Grade 12
Participant 16	Male	29	African	African traditional religion	In a relationship	Traditionally circumcised	Grade 9
Participant 17	Male	37	African	African traditional religion	In a relationship	Uncircumcised	Grade 11
Participant 18	Male	29	African	Old Testament Christian	In a relationship	Uncircumcised	College diploma
Participant 19	Male	39	African	African traditional religion	In a relationship	Traditionally circumcised	Grade 10
Participant 20	Male	41	African	Old Testament Christian	Married	Uncircumcised	No formal education

**Table 2.** Summary of the Main Categories/Themes of Description.

Conceptions of VMMC	Understanding of VMMC	Experiences of VMMC
Category 1 Medical circumcision as a sin	Category 1 VMMC and HIV prevention	Category 1 Negative secondary experiences
Category 2 VMMC as unnatural	Category 2 Traditional versus medical circumcision	Category 2 Positive secondary experiences
Category 3 Unknown and unnecessary	Category 3 Medical circumcision time and healing time	Category 3 No experiences

The purpose of this study was to analyze the qualitative differences in Zulu men’s experiences, understanding, and conceptions of VMMC in KZN. The lens of phenomenographic enquiry was used to examine differences in the interviews by focusing on patterns of thought, language, values, and behavior and the meaning that participants associated with this in relation to VMMC. The results of the data analysis led to the identification of three distinct, yet interrelated categories of differences in experiences, understanding, and conceptions of VMMC, totaling nine categories of differences.

### Conceptions of VMMC

Participants’ conceptions of VMMC were also elicited through a set of semistructured questions which related to personal definitions of VMMC, personal opinions, and beliefs regarding VMMC.

#### Medical Circumcision a “Sin”

It appears as though participants conceive VMMC as something that is sinful. Participants’ constant reference to religion to justify why they thought it was wrong is indicative of this. The following statements were made by participants in this regard:

*“What I tell myself is that God would not have created me with a piece of flesh that he would want removed.” (Participant18)*

*“God would not create something if he wanted a man to remove it and also the Bible its stating that a person with a missing portion of his front [penis] will not enter the kingdom of heaven. Even my father always tells us this so; therefore, I can’t go against God and my father here on earth.” (Participant 8)*

#### VMMC as Unnatural

Participants also seem to think that performing circumcision is unnatural. Some feel that a part of their body becomes lost or missing forever and the remaining scar is a constant reminder of how they have defiled their bodies. To other participants, medical circumcision is unnatural because the way in which it is performed is different from the traditional circumcision that they are historically aware of. In addition, the act of VMMC could also be seen as unnatural in that it is perceived to affect sexual functioning and individual functioning and identity as a man. These participants are men in the 45–49 age group whose beliefs and upbringing are strongly rooted in past traditional practices. According to these males, circumcision has connotations of masculinity, while also respecting the manner in which the body of a man is naturally created. This was supported by the following statements:

*I don’t think it would be right for me to have my foreskin removed because I believe it’s doing something. If it was not meant to be there, then it won’t naturally be there but since I have been created with it, it will be very wrong of me to take out what was original in the eyes of God. (Participant 20)*

*Im not having part of my penis chopped off. . .no ways, then how am I supposed to function nicely as a man when part of my manhood is missing? (Participant 3)*

#### Unknown and Unnecessary

Four participants reported not having any information about the nature or benefits of VMMC. These participants stated that they had never heard of circumcision and therefore saw it as unnecessary. These participants also had low levels of education and were living in extremely remote rural areas. The excerpts below support this category of description:

*I know that circumcisions are done but I have not got information about why they are done. (Participant 3)*

*I’m not interested in circumcision because I am a spiritual person who is not really that much into women so I don’t think I need this thing. (Participant 15)*

### Understanding of VMMC

Zulu men’s understanding of VMMC was also attained through a semistructured interview guide with questions relating to participants’ understanding of the procedure in terms of HIV prevention, healing time, benefits, and differences between traditional and medical approaches.

#### VMMC and HIV Prevention

Fifteen participants seemed to be aware that VMMC provides some form of protection against HIV infection; however, participants were not aware of the degree of protection it provides. A significant number of participants reported having acquired this information by word of mouth and although they responded in this way, it may appear that some were not truly convinced of this reality and had therefore opted not to be circumcised medically. This was supported by the following statements:

*The chances of getting infected with HIV/AIDS are significantly reduced. So are the chances of getting infected with other associated sexually transmitted infections. (Participant 2)*

*I have heard people saying that if you are circumcised, you are prevented from many diseases like HIV because of the dirt that is staying within the foreskin so by cutting it off the chances of being infected become low. (Participant 12)*

#### Traditional Versus Medical Circumcision

Participants were aware of the nature of both procedures but were not convinced as to which was better. Ten participants had the understanding that both procedures will lead to one outcome—the removal of the foreskin, irrespective of how this was performed. These participants also reported that medical circumcision defied the laws of traditional circumcision because of the manner in which it was performed. This was supported by the statements below:

*To me it does not matter where and how you do the circumcision, the end result is the same. (Participant 16)*

*I think that there is no major difference between the two - it [is] just that one is using [a] modern method and the other one is taking a person’s tradition into account, but all in all they are dealing with the private part of a male to make it better. (Participant 11)*

*I have heard people talking about this medical circumcision. I have found that it’s different from what we used to do back in the day. You see what we as Zulu people used to do was cut the vein of the penis using a thorn to make you a proper man who can function well in bed. What they are doing today, cutting off [the] foreskin. . .an entire part of a person, that is new to me. These modern things are causing problems because now you are left with a missing part of your thing there in front and then also how are you going to do the traditional thing when this modern thing has interfered first. This is not our culture and because of this we are losing our practices. (Participant 19)*

The other participants were aware that the traditional practice came with complications relating to pain, bleeding, and infection because of the nature in which it was performed. It is, however, interesting to note that although these participants have responded in such a way, they are still included among those who were uncircumcised.

*I have heard about the mountain circumcision method on the radio, where they are saying that 20 people had died from it. So I felt like it is not good. The medical circumcision method that is done at the hospital is better than this one. (Participant 14)*

*Medical circumcision is better because people are safe. They are provided with the necessary care. (Participant 4)*

#### Medical Circumcision Time and Healing Time

Participants revealed that the time for performing VMMC was age-related and healing time is influenced by the age that you are at the time of the procedure. According to most participants, VMMC was something that was for the modern teenage boy.

*I think that the best time for circumcision is when the boy is very young, at least about 12 years old because everything is still soft. He will get cut easily and will heal very quickly. (Participant 8)*

*The earlier, the better because I see most small (boys) doing it and they don’t have complications like the older men. (Participant 13)*

### Secondary Experiences of VMMC

In order to elicit Zulu men’s experiences of VMMC, a set of guiding interview questions were developed and used with constant probing to attain a more in-depth perspective in this regard.

#### Negative Experiences and Perceptions

Nine participants in this study reported experiences that were not pleasant regarding VMMC which contributed to a generally negative perception of VMMC and a lack of desire to undergo the procedure. These experiences stemmed from conversations and close interactions with individuals who had undergone the procedure. These experiences were related to the nature of the procedure, during and postoperatively. The following statements serve to support this category:

*I was waiting in the clinic line about to go for circumcision when I heard one of the boys inside screaming. I got very scared and, what was worse, was seeing how the boys were walking as they were coming out of the room. . . I thought no, I won’t be able to handle this. (Participant 5)*

*See one of my friends had done this and was complaining of pain especially at night because he could not control getting an erection, he was always complaining of this problem and the stitches were cracking, making it worse with bleeding and all of that. (Participant 4)*

#### Positive Experiences and Perceptions

Seven participants reported no problems with VMMC and had not reported any bad experiences about the procedure from anyone within their immediate sphere of contact. However, this group of participants either had a general fear because of not having enough adequate information about the procedure, or had simply not contemplated undergoing VMMC.

*“My son had undergone circumcision and had no problems; he healed quickly and was back to his normal self, even playing, in like about one week.” (Participant 17)*

*“I know quite a lot of people who have done this and have no complaints, it [is] just that I am generally afraid. Sometimes I think I may not be as lucky as them not to feel any pain or experience complications.” (Participant 7)*

#### No Experiences of VMMC

Four participants who reported not knowing anything about VMMC in any form whatsoever. These participants had also not been acquainted with anyone who spoke of VMMC, let alone anyone who had undergone it.

*“I have heard of small boys going for it but have never heard of what it involves or why they are doing it.” (Participant 11)*

*“There is no experience I have with medical circumcision because there is no one I know who has done [it] and also here it’s something that I don’t really hear people talking about. (Participant 9)”*

## Discussion

This study analyzed Zulu men’s conceptions, understanding, and experiences of VMMC in KZN. A sample of 20 uncircumcised men between the ages of 18 and 49 formed part of the study. The conceptions, understanding, and experiences of Zulu men regarding VMMC were diverse. These were grouped into three categories of description respectively, thus making a total of nine categories which were all interconnected.

Participants’ conceptions of VMMC were shaped both by religion and culture together with the associated experiences and understanding thereof. Zulu men’s view of VMMC as a sin can be associated with early Christian Church beliefs. This poses a possible barrier to access and utilization of the service. The differences in practice of traditional and medical circumcision reported by participants’ show how culture and tradition influence how they understand and conceptualize VMMC in their minds. According to Vincent (2008), culture, religion, and ethnic identity are a concern with regards to circumcision because those who practice it, do so as a form of ethnic identity and those who do not, also do this as a part of an ethnic identity.

In a study by Golomski and Nyawo (2017), it was reported that traditionalist African churches rejected the notion of VMMC, while Pentecostal Charismatic Churches were seen to be more likely to accept it as a procedure due to metaphysical beliefs that circumcision allowed one to receive God’s grace. Milford et al. (2012) argue that engagement with the religious and cultural practices associated with circumcision is necessary to ensure successful implementation of the VMMC program.

Certain men viewed VMMC as unnatural, citing concerns related to functionality of their penis and overall appearance. This finding is in line with the results of studies conducted on sexual functioning and male circumcision. The findings suggested that male circumcision resulted in a decrease in masturbatory pleasure and sexual enjoyment, indicating that circumcision affected sexual functioning in men (Bronselaer et al., 2013; Kim & Pang, 2007). Other studies found that adult male circumcision did not affect sexual functioning or overall performance (Kigozi et al., 2008; Krieger et al., 2008). The differences in these findings could be attributed to the data collection methods, sample size, and context in which data were collected.

Certain Zulu men also reported that VMMC was unnecessary, while others did not have sufficient information about the nature of the procedure in order to make an informed decision. The reasons for this view are similar to those reported by Hatzold et al. (2014) who found that men did not want to be circumcised because they perceived themselves to be at a low risk of attaining HIV infections. This highlights the need for providing accurate and detailed information about the benefits of VMMC and the nature of the procedure. Evens et al. (2014) suggest that men should be given information about what to expect before, during, and after the procedure; this information should typically be incorporated into demand creation interventions.

The participants’ understanding of VMMC was in relation to HIV prevention, differences between medical and traditional circumcision and VMMC performance and healing time. Although males had an understanding of the partial protection provided by VMMC, the degree of partial protection was not explicitly communicated to the researcher, despite several attempts at probing to attain this understanding. This could point to a lack of insight of the benefits of VMMC. These findings concur with those of Kelly et al. (2012) who also found that men had limited knowledge and understanding in terms of HIV and STI acquisition. Many men were of the perception that the removal of the foreskin which harbored dirt is what assisted in the HIV and STI prevention.

A study by L’Engle et al. (2014) showed that men had a good understanding of the partial protection offered by VMMC as these participants were able to describe how circumcision reduced HIV transmission and the intention to use other HIV protective measures such as practicing safe sexual intercourse. Similar findings were reported in a later study by Kibira et al. (2017). The difference in such findings may be attributed to the levels of education among interviewed participants, the general practice of circumcision, and the cultural context. In this present study, the cultural context and historical traditions of circumcision heavily influence beliefs and knowledge passed on regarding circumcision. The information about the benefits of VMMC is challenged by cultural norms. Mobilization for uptake should, therefore, take these cultural factors into consideration.

The understanding of traditional and medical circumcision was also varied, with a significant number of participants revealing that no difference existed between the two procedures. This finding is significant because it serves to highlight the degree to which certain males may be misinformed about the nature and outcomes of an intervention like VMMC. A deeper probing of this area also revealed that Zulu men who had this understanding preferred traditional circumcision over medical circumcision because of the meaning that was associated with this method of circumcision and the way in which it had been performed historically. These findings demonstrate the tensions that exist between modern and traditional practices. It is these tensions that pose as barriers to the utilization of health services including VMMC. Howard-Payne and Bowman (2017) state that medicalizing circumcision may result in tension, in that the meanings associated with the two approaches differ, particularly in terms of masculinity and the traditional rite of passage to manhood. The main element of traditional circumcision, for instance, is endurance of pain and undergoing various tests to prove one’s manhood (Mavundla et al., 2009). The surgically performed circumcision which uses local anesthetic and other pain-relieving agents, in this case, undermines the value of this procedure from a historical, traditional, and cultural perspective (Yaser & Khauli, 2007).

The difference between traditional and medical circumcision was understood in terms of the perceived nature and context of both procedures. Participants’ understanding of medical circumcision could be associated with both knowledge and assumption of the scientific nature and inherent safety of the procedure, while the understanding of traditional circumcision which was associated with ill health and potential complication could be attributed to negative experiences and complications reported during ritual circumcision. Bailey et al. (2008) reported that men undergoing traditional male circumcision were almost twice as likely to experience complications as opposed to those who undergo medical circumcision. An earlier study by Mogotlane et al. (2004) found that restriction in fluid intake and the generally poor hygienic conditions in which traditional circumcision was performed often resulted in local and respiratory infections and poor wound healing.

In terms of medical circumcision time and healing time, participants seem to believe that only young boys, because they were perceived to heal quicker, should undergo the procedure. Other participants believed that since the nature of the procedure does not allow for one to experience pain, then one does not transition to manhood since an integral part of attaining manhood is the ability to withstand pain while the ritual is conducted. This, yet again, could explain why certain men choose not to access VMMC services. Humphries et al. (2015) postulate that masculinity and male sexual identity are interlinked and possibly have an influence on the uptake of VMMC; an understanding of these factors and how they manifest in various contexts is thus crucial to overcoming this as a barrier to uptake. In another study on the perceptions of VMMC in traditionally circumcising and non-circumcising communities, Rennie et al. (2015) found that tensions between traditional and medical circumcision practices could account for the poor uptake of VMMC.

Participants reported negative and positive secondary experiences of VMMC and also no experiences pertaining to the procedure. Negative experiences about VMMC were related to the postoperative complications of pain and poor wound healing experienced and communicated by peers and other close influencers. These negative secondary experiences are a reflection of the perception that these men have adopted with regard to VMMC. The apprehension of pain and other postoperative complication has been cited in other studies (Moyo et al., 2015; Rennie et al., 2015; Rupfutse et al., 2014).

Positive experiences stemmed mostly from witnessing a biological child or close relative in the family undergoing VMMC. Despite these experiences, these men still verbalized fears about the procedure, probably stemming from a general fear of the unknown or perceptions related to pain and other complications.

Participants who reported not having any experiences with VMMC were not acquainted with anyone who had undergone the procedure. It is interesting to note that these males were mainly from deep rural settings with minimal, to no secondary or tertiary level, education. Research on the acceptability of medical circumcision has shown that in certain settings, men with higher levels of education are more likely to be willing to undergo VMMC (Nyaga et al., 2014; Spees et al., 2019). The geographical location of males is also significant since resource-constraint settings often mean that individuals lack access to services and dissemination of information about general health matters also becomes compromised.

## Limitations

The study was limited in the sample size and in that a qualitative approach was used. The finding cannot, therefore, be generalized to all males in the KZN province of South Africa. However, the study adds to the body of literature in that it provides greater knowledge on how VMMC is understood in a setting in which tradition and culture heavily influence the uptake of VMMC.

## Conclusion

The results of the study show that conceptions, understanding, and experiences of Zulu men regarding VMMC are intertwined, and are heavily influenced by culture, tradition, and religion. Negative experiences relating to postoperative complications and a generally poor understanding of the nature of VMMC could account for the poor uptake of VMMC among males in the studied age group. The major contribution of this study is that it provides deeper awareness of how Zulu men conceptualize and understand VMMC with the lens of religion, culture, and tradition. Although the concept of religion, culture, and medical circumcision has been explored in previous studies, the context within which this has been studied is different. Moreover, the conception of circumcision as a sin among Zulu men has not been cited and thus has implications for the type of messaging to be provided during demand generation. Facilitating the uptake of VMMC among Zulu men may thus be reliant on the provision of tailored messaging about VMMC that takes into account the religious, cultural and traditional dynamics. Provision of tailored education is reliant upon community-level engagement with key traditional and religious figures.

Tailored messaging taking into account the religious, traditional, and cultural dynamics of Zulu men could include the following messages:

(1) VMMC is one of the oldest surgical procedures performed worldwide for medical, religious, and cultural reasons.
(2) Foreskin removal done during medical circumcision promotes improved penile hygiene and cleanliness, which is closer to Godliness.
(3) Medical male circumcision does not impair or compromise one’s functioning as a man, instead one’s health may be improved as it provides partial protection against HIV infection.
(4) Undergoing VMMC has health benefits beyond just partial protection against HIV and other sexually transmitted infections. Other benefits include decreased chances of attaining penile cancers.
(5) Part of manhood involves making positive health choices and responsible sexual health behaviors—medical circumcision is a step toward this direction as it has indirect benefits for one’s female counterpart.

## Supplemental Material

Data_collection_instruments_AJMH – Supplemental material for Zulu Men’s Conceptions, Understanding, and Experiences of Voluntary Medical Male Circumcision in KwaZulu-Natal, South Africa Click here for additional data file. Supplemental material, Data_collection_instruments_AJMH for Zulu Men’s Conceptions, Understanding, and Experiences of Voluntary Medical Male Circumcision in KwaZulu-Natal, South Africa by Celenkosini Thembelenkosini Nxumalo and Gugu Gladness Mchunu in American Journal of Men’s Health

DOH_Approval_Letter_(Nxumalo) – Supplemental material for Zulu Men’s Conceptions, Understanding, and Experiences of Voluntary Medical Male Circumcision in KwaZulu-Natal, South Africa Click here for additional data file. Supplemental material, DOH_Approval_Letter_(Nxumalo) for Zulu Men’s Conceptions, Understanding, and Experiences of Voluntary Medical Male Circumcision in KwaZulu-Natal, South Africa by Celenkosini Thembelenkosini Nxumalo and Gugu Gladness Mchunu in American Journal of Men’s Health

Ethical_approval – Supplemental material for Zulu Men’s Conceptions, Understanding, and Experiences of Voluntary Medical Male Circumcision in KwaZulu-Natal, South Africa Click here for additional data file. Supplemental material, Ethical_approval for Zulu Men’s Conceptions, Understanding, and Experiences of Voluntary Medical Male Circumcision in KwaZulu-Natal, South Africa by Celenkosini Thembelenkosini Nxumalo and Gugu Gladness Mchunu in American Journal of Men’s Health

Letter_confirming_editing – Supplemental material for Zulu Men’s Conceptions, Understanding, and Experiences of Voluntary Medical Male Circumcision in KwaZulu-Natal, South Africa Click here for additional data file. Supplemental material, Letter_confirming_editing for Zulu Men’s Conceptions, Understanding, and Experiences of Voluntary Medical Male Circumcision in KwaZulu-Natal, South Africa by Celenkosini Thembelenkosini Nxumalo and Gugu Gladness Mchunu in American Journal of Men’s Health

Sample_transcriptfor Zulu Men’s Conceptions, Understanding, and Experiences of Voluntary Medical Male Circumcision in KwaZulu-Natal, South Africa Click here for additional data file. Sample_transcript for Zulu Men’s Conceptions, Understanding, and Experiences of Voluntary Medical Male Circumcision in KwaZulu-Natal, South Africa by Celenkosini Thembelenkosini Nxumalo and Gugu Gladness Mchunu in American Journal of Men’s Health This article is distributed under the terms of the Creative
Commons Attribution 4.0 License ( http://www.creativecommons.org/licenses/by/4.0/) which
permits any use, reproduction and distribution of the work without
further permission provided the original work is attributed as specified
on the SAGE and Open Access pages ( https://us.sagepub.com/en-us/nam/open-access-at-sage).
