# Supplementary material for: Zulu Men’s Conceptions, Understanding, and Experiences of Voluntary Medical Male Circumcision in KwaZulu-Natal, South Africa
Source: Am J Mens Health. 2020 Mar 5;14(2):1557988319892437. doi: 10.1177/1557988319892437 (PMC7059234; doi:10.1177/1557988319892437)
Supplement: Data_collection_instruments_AJMH – Supplemental material for Zulu Men’s Conceptions, Understanding, and Experiences of Voluntary Medical Male Circumcision in KwaZulu-Natal, South Africa [file Data_collection_instruments_AJMH.docx]

**DATA COLLECTION INSTRUMENT**

**Title of research project: An analysis of Zulu men’s conceptions, understanding and experiences of Voluntary Medical Male Circumcision in KwaZulu-Natal, South Africa**

**DEMOGRAPHIC DATA**

| AGE |  | | | | | | | | | | | | | |
| --- | --- | --- | --- | --- | --- | --- | --- | --- | --- | --- | --- | --- | --- | --- |
| **GENDER** | | | | | | | | | | | | | | |
| FEMALE | | | | | | | | | |  | MALE | |  | |
| ETHNIC GROUP | | |  | | | | | | | | | | | |
| RELIGIOUS/CULTURAL BELIEF | | | | | | |  | | | | | | | |
| **RELATIONSHIP STATUS** | | | | | | | | | | | | | | |
| SINGLE | |  | | MARRIED | | | |  | IN A RELATIONSHIP | | | | |  |
| CIRCUMCISED | | | | |  | UNCIRCUMCISED | | | | | |  | | |
| **HIGHEST LEVEL OF EDUCATION** | | | | | | | | | | | | | | |
|  | | | | | | | | | | | | | | |

**GUIDING INTERVIEW QUESTIONS**

**Title of research: An analysis of Zulu men’s conceptions, understanding and experiences of Voluntary Medical Male Circumcision in KwaZulu-Natal, South Africa**.

**QUESTIONS FOR UNCIRCUMCISED MALES**

*(A) Conceptions regarding VMMC.*

1. How would you define medical male circumcision?

2. From talking to others what have you heard about circumcision?

3. What is your personal opinion and belief regarding medical male circumcision?

4. In your view why is circumcision necessary?

5. Explain when do you think is the right time to circumcise?

*(B)Understanding regarding VMMC*

1. In your own understanding, what would be the benefits of circumcision?

2. What is your understanding of VMMC in terms of the healing time?

3. What is your understanding of medical male circumcision in relation to HIV prevention?

4. In your understanding, explain the difference between traditional male circumcision and medical male circumcision.

*(C) Experiences regarding VMMC*

1. Explain what influenced your decision not to undergo male circumcision?

2. What is your personal experience regarding medical male circumcision?

3. Explain why you would or would not recommend that males undergo medical male circumcision.
