# Supplementary material for: Zulu Men’s Conceptions, Understanding, and Experiences of Voluntary Medical Male Circumcision in KwaZulu-Natal, South Africa
Source: Am J Mens Health. 2020 Mar 5;14(2):1557988319892437. doi: 10.1177/1557988319892437 (PMC7059234; doi:10.1177/1557988319892437)
Supplement: DOH_Approval_Letter_(Nxumalo) – Supplemental material for Zulu Men’s Conceptions, Understanding, and Experiences of Voluntary Medical Male Circumcision in KwaZulu-Natal, South Africa [file DOH_Approval_Letter_(Nxumalo).pdf]

health

Department:
Health
PROVINCE OF KWAZULU-NATAL

DIRECTORATE:

Physical Address: 330 Langalibalele Street, Pietermaritzburg
Postal Address: Private Bag X9051
Tel: 033 395 2805/ 3189/ 3123 Fax: 033 394 3782
Email: [illegible]
www.kznhealth.gov.za

Health Research & Knowledge Management

Ref: KZ_201811_007

Dear Mr T C Nxumalo
(UKZN)

<u>Subject: Approval of a Research Proposal:</u>

1. The research proposal titled **'An analysis of Primary Health Care stakeholders' experiences, understanding and conceptions of Voluntary Medical Male Circumcision (VMMC) in KZN: A Phenomenographic study'** was reviewed by the KwaZulu-Natal Department of Health (KZN-DoH).

   The proposal is hereby **approved** for research to be undertaken at the selected sites at KZN-DoH.

2. You are requested to take note of the following:

   a. *Kindly liaise with the facility manager BEFORE your research begins in order to ensure that conditions in the facility are conducive to the conduct of your research. These include, but are not limited to, an assurance that the numbers of patients attending the facility are sufficient to support your sample size requirements, and that the space and physical infrastructure of the facility can accommodate the research team and any additional equipment required for the research.*
   b. *Please ensure that you provide your letter of ethics re-certification to this unit, when the current approval expires.*
   c. *Provide an interim progress report and final report (electronic and hard copies) when your research is complete.*

3. Your final report must be posted to **HEALTH RESEARCH AND KNOWLEDGE MANAGEMENT, 10-102, PRIVATE BAG X9051, PIETERMARITZBURG, 3200** and e-mail an electronic copy to hrkm@kznhealth.gov.za

For any additional information please contact Ms G Khumalo on 033-395 3189.

Yours Sincerely

Mr J Govender
Chief Director: Health Service Planning, Monitoring & Evaluation
Date: 23/11/18
