# Supplementary material for: Zulu Men’s Conceptions, Understanding, and Experiences of Voluntary Medical Male Circumcision in KwaZulu-Natal, South Africa
Source: Am J Mens Health. 2020 Mar 5;14(2):1557988319892437. doi: 10.1177/1557988319892437 (PMC7059234; doi:10.1177/1557988319892437)
Supplement: Ethical_approval – Supplemental material for Zulu Men’s Conceptions, Understanding, and Experiences of Voluntary Medical Male Circumcision in KwaZulu-Natal, South Africa [file Ethical_approval.pdf]

UNIVERSITY OF KWAZULU-NATAL ™
INYUVESI YAKWAZULU-NATALI

10 December 2018

Mr CT Nxumalo (215079134)
School of Nursing and Public Health
College of Health Sciences
Thembz92@gmail.com

Dear Mr Nxumalo

Protocol: An analysis of primary health care stakeholders experiences, understanding and conceptions of voluntary medical male circumcision (VMMC) in KZN: A phenomenographic study.
Degree: PhD
BREC REF: BE627/18

A sub-committee of the Biomedical Research Ethics Committee has considered and noted your application received on 10 October 2018.

The study was provisionally approved pending appropriate responses to queries raised. Your response received on 27 November 2018 to BREC letter dated 24 October 2018 has been noted by a sub-committee of the Biomedical Research Ethics Committee. The conditions have been met and the study is given **full ethics approval**. Please ensure that site permissions are obtained and forwarded to BREC for approval before commencing research at a site.

This approval is valid for one year from **10 December 2018.** To ensure uninterrupted approval of this study beyond the approval expiry date, an application for recertification must be submitted to BREC on the appropriate BREC form 2-3 months before the expiry date.

Any amendments to this study, unless urgently required to ensure safety of participants, must be approved by BREC prior to implementation.

Your acceptance of this approval denotes your compliance with South African National Research Ethics Guidelines (2015), South African National Good Clinical Practice Guidelines (2006) (if applicable) and with UKZN BREC ethics requirements as contained in the UKZN BREC Terms of Reference and Standard Operating Procedures, all available at http://research.ukzn.ac.za/Research-Ethics/Biomedical-Research-Ethics.aspx.

BREC is registered with the South African National Health Research Ethics Council (REC-290408-009). BREC has US Office for Human Research Protections (OHRP) Federal-wide Assurance (FWA 678).

The sub-committee's decision will be noted by a full Committee at its next meeting taking place on 11 December 2018.

We wish you well with this study. We would appreciate receiving copies of all publications arising out of this study.

Yours sincerely

Professor V Rambiritch
Chair: Biomedical Research Ethics Committee

Supervisor: mchunug@ukzn.ac.za
Postgrad admin: Carol Dhanraj

Biomedical Research Ethics Committee
Professor V Rambiritch (Chair)
Westville Campus, Govan Mbeki Building
Postal Address: Private Bag X54001, Durban 4000
Telephone: +27 (0) 31 260 2486 **Facsimile:** +27 (0) 31 260 4609 **Email:** brec@ukzn.ac.za
Website: http://research.ukzn.ac.za/Research-Ethics/Biomedical-Research-Ethics.aspx
