# Supplementary material for: Zulu Men’s Conceptions, Understanding, and Experiences of Voluntary Medical Male Circumcision in KwaZulu-Natal, South Africa
Source: Am J Mens Health. 2020 Mar 5;14(2):1557988319892437. doi: 10.1177/1557988319892437 (PMC7059234; doi:10.1177/1557988319892437)
Supplement: Letter_confirming_editing – Supplemental material for Zulu Men’s Conceptions, Understanding, and Experiences of Voluntary Medical Male Circumcision in KwaZulu-Natal, South Africa [file Letter_confirming_editing.docx]

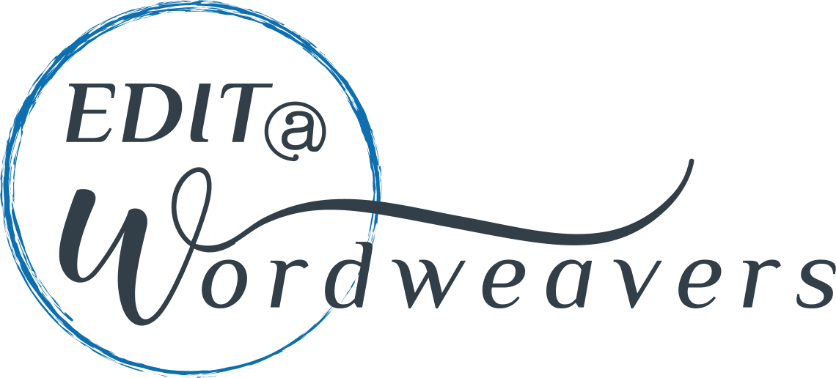


A collaboration under the Wordweavers cc umbrella/CK 2006/156780/23

18 July 2019

Submissions

The American Journal of Men’s Health

**LETTER CONFIRMING EDITING OF ARTICLE FOR SUBMISSION: *Zulu men’s conceptions, understanding and experiences of voluntary medical male circumcision in KZN* BY Mr THEMBA NXUMALO**

This letter serves to confirm that this article was edited by the above collaboration in the person of Mr Govin Reddy, and that an editing report was created which indicated to Mr Nxumalo the areas of the article which did not confirm to the submission requirements of the journal in terms of structure, layout and formatting and style.

The article was also edited for language, syntax, spelling, grammar, tense and punctuation and some discrepancies in referencing between citations and the reference list were also brought to light.

The article was returned to Mr Nxumalo with the suggested corrections which he was to make prior to submission to your journal.

We trust that the article will prove satisfactory.

Kind regards

**Catherine Eberle**


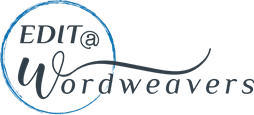


Email: info@editatwordweavers.co.za
