# Supplementary material for: Zulu Men’s Conceptions, Understanding, and Experiences of Voluntary Medical Male Circumcision in KwaZulu-Natal, South Africa
Source: Am J Mens Health. 2020 Mar 5;14(2):1557988319892437. doi: 10.1177/1557988319892437 (PMC7059234; doi:10.1177/1557988319892437)
Supplement: Sample_transcriptfor Zulu Men’s Conceptions, Understanding, and Experiences of Voluntary Medical Male Circumcision in KwaZulu-Natal, South Africa [file Sample_transcript.docx]

I: Interviewer

R: Respondent.

I: Ok, ngicela ukuzi-introducer mina kuqala. Igama lami ngiwu Themba wakwa Nxumalo. Ngiwu-nurse plus futhi ngenza ucwaningo ngokusoka. Kukhona into engifuna ukukubuza yona-ke ngakho ukusoka, ngifuna ukwazi ukuthi ucabangani ngayo, wena okokuqala nje awusokile?

**I: Ok. I would like to introduce myself first. My name is Themba Nxumalo. I am a nurse plus I am doing research on circumcision. There is something that I would like to ask you about circumcision. I want to know what you think about it. Firstly, you are not circumcised, are you?**

R: Neh?

**R: No**

I: Awusokile angithi?

**I: You are not circumcised, right?**

R: Yah, angisokile.

**R: Yes. I am not circumcised.**

R: Ehhe

**R: Yes**

I: Uh, waqala nini ukuzwa ngendaba yokusoka, wawuneminyaka emingaki?

**R: How old were you when you first heard about circumcision?**

R: Uh, mhlampe 13.

**R: Um, maybe 13.**

I: 13, ehhe, between 13 ngoba usuna 18 manje, from 13 wezwa kanjani ngako?

**I: 13, yes, between 13 and you are now 18. When you were 13 how did you hear about it?**

R: Ngezwa laphana kuthiwa bayo [Inaudible: 09:01:34] manje eyi angazi ibhadi lingalana kimi, manje ngiyesaba mina ukusoka.

**R: I heard there. They said they were [inaudible] and I do not know why I have bad luck. I am afraid of getting circumcised.**

I: Sengisholo ukuthi uqala ukuzwa ngayo, ngiseza, ngizoza lapho, ngiyeza lapho vese kuleyondaba yokuthi indaba ungathandi but sengisholo, ukuqala ukuzwa ngokusoka, wezwa ngobani kuthiwani?

**I: I mean when you first heard about it. I am still coming to that issue of why you do not like it. I am still on how you first heard about circumcision and what was being said about it.**

R: Hhayi ngezwa esikoleni nje kuthiwa kuyasokwa [Inaudible: 09:01:53]

**R: I heard about it at school. They said there is circumcision that is being done [inaudible].**

I: Mm, nani enye? Basho lokho kuphela?

**I: Mm, what else? Is that all they said?**

R: Eyi, okunye sengikukhohliwe, ngangifunda e-primary.

**R: I have forgotten the other thigns that they said. I was still in primary school then.**

I: Kwakufike bani esikoleni ezonitshela lokho?

**I: Who had come to school to tell you about that?**

R: Kwakufike, abanye nje abantu, ngathi basebenza esibhedlela vele.

**R: Some people had come. I think they worked at the hospital.**

I: Ok.

**I: Ok.**

R: Bafika ngemoto e-yellow, khona le ejwayele ukuthatha izingane uma ziyosoka.

**R: They arrived in a yellow vehicle. There is this vehicle that picks up kids when they are going to get circumcised.**

I: Ehhe, so manje 13 to 18 years yini ekuvimbile ukuthi ungathandi ukusoka?

**I: Yes, so now 13 to 18 years, what is preventing you from getting circumcised?**

R: Hhayi ayikho.

**R: There is nothing.**

I: Kodwa phela awenzile nje?

**I: But you have not been circumcised.**

R: Angenzanga vele.

**R: True, I have not been circumcised.**

I: Ehhe so, kukhona into ekuvimbili I am sure noma into oyizwile eyenze ukuthi ungasoki, yini leyo?

**I: Yes, so there is something that is preventing you from doing it I am sure or perhaps it is something that you heard that is preventing you from going to get circumcised?**

R: Eyi ukuthi bakusoka ngani laphana angazi noma i [Inaudible: 09:02:52]

**R: I do not know what they use when they perform the circumcision procedure [inaudible].**

I: Mm, yini, angizwa, bakusoka kambi?

**I: Mm, I beg your pardon?**

R: Ehhe, ngezwa kuthiwa basoke kambi.

**R: Yes, I heard they were circumcised badly.**

I: Wezwa ngobani?

**I: Who did you hear that from?**

R: Ngezwa esikoleni, khona omunye umjita esihlala naye endaweni, bamusoka waphinde wabuyela.

**R: I heard at school. There is one guy that we live in the same area with. They circumcised him and then he had to return to be circumcised again.**

I: Bamusoka kabi kanjani mhlasimpe?

**I: They circumcised him badly in what way?**

R: Ngingathini, eyi, angazi ngoba wala ukungitshengisa kodwa wahinde wabuyela esibhedlela bamusika kabusha, ngathi aba-cut-anga kahle.

**R: What can I say? I do not know because he refused to show me. However, he returned to the hospital and he was circumcised again. It seemed as if they had not been cut correctly initially.**

I: Mm.

**I: Mm**

R: Ukuyo-cut-isisa.

**R: So they had to correct the cutting.**

I: Oh, ok. So, from that time wena uzwa lokho, kwakumuphi unyaka uzwa lokho?

**I: Oh ok. So when was it when you heard that? what year was it when you heard that?**

R: ngicinga unyaka olandela ophelile.

**R: I think it was two years ago.**

I: Mm, ok. So, wena ngokwakho umuntu ngeke uze umgqugquzele ukuthi asoke?

**I: Mm, ok. So personally would you encourage a person to get circumcised?**

R: Hhayi ngeke.

**R: No, I would not.**

I: Ngobani?

**I: Why not?**

R: Ngobani?

**R: Why not?**

I: Mm.

**I Mm**

R: ngoba akulunganga ukusoka.

**R: Because circumcision is bad.**

I: Akulunganga?

**I: It is bad?**

R: Hhayi akulunganga.

**R: It is bad.**

I: yini ndaba?

**I: Why?**

R: Hhayi kumane kube ibhadi lakho ufe.

**R: You could die if you are unlucky.**

I: Mm, no, webhuti ngicela ukhulume ukhululeke uyabo lokho ozobe ukusho la kuzobe kusisiza nathi ukuthi sithole ukuthi ene ikuphi la esinga-improve-a khona ukwenza, uyayizwa leyonto? Yah ungaqhubeka ukhulume bengikulalele.

**I: Mm, no, please speak freely because what you will be saying hear will help us to find out where we can improve, do you understand? Ok, continue talking. I was listening.**

R: Sengiqedile.

**R: I am done.**

I: Ilokho kuphela. Ok, ngokuzwa kwakho-ke ukusoka kusiza kuphi, umuntu kumsiza kephi, ngokuzwa kwakho?

**I: That is all. Ok, according to what you have heard what are the benefits of circumcision?**

R: Kumsiza ezifweni ukuthi ungazitholi kalula.

**R: It helps with prevention of infection from diseases.**

I: Izifo ezinjengani?

**I: What kind of dieases?**

R: HIV.

**R: HIV**

I: Mm.

**I: Mm**

R: Ezinye angizazi-ke, ngiyazikhohlwa.

**R: I do not know the other diseases. I forget them.**

I: Ok, umuntu uma esokile kumthatha isikhathi esingakanani ukuthi aphole?

**I: Ok. How long does it take for a circumcised person to completely heal?**

R: Angazi noma 2 weeks noma 7 days.

**R: I do not know whether it takes two weeks or seven days.**

I: Mm, ok. Leyo information uyazelaphi, uyithathe kuphi?

**I: Mm ok. Where did you get that information from?**

R: Basitshela esikoleni.

**R: They told us at school.**

I: Ok, uyawazi umehluko between ukusoka lokhu, ngendlela yase-clinic nalokhu soka lokhu kwasentabeni.

**I: Ok. Do you know the difference between medical male circumcision and traditional male circumcision?**

R: Eyi angazi.

**R: I do not know it.**

I: Ngokucabanga kwakho kuyafana noma akufani?

**I: Do you think it is the same or not?**

R: Eish, angeke kufane.

**R: I do not think it will be the same.**

I: Kuhluka kuphi?

**I: Where does it differ?**

R: Mhlampe entabeni abanayo lemijovo abaqale bakujove ngayo.

**R: Maybe at the mountain they do not have these injections that you are first injected with.**

I: Mm.

**I: Mm**

R: And ngizwa kuthiwa bajova ngocelemba lapha, angazi.

**R: And I have heard that they are cut with a bush knife there. I do not know.**

I: Uzwe ngobani?

**I: Who told you that?**

R: Ngezwa nje esikoleni.

**R: I heard about it at school.**

I: Hhayi ok, ngiyakuzwa-ke. Uh zikhona izinkolelo onazo mayelana nokusoka njengoba nje uthi ukholelwa kokwesintu, ngokwesintu ithini inkolo yakho ngokusoka?

**I: Ok, I hear you. Do you have any beliefs about circumcision? You have mentioned that you believe in African traditions. What do African traditions say about circumcision?**

R: Ithi asisoki isintu.

**R: It says we do not practice circumcision.**

I: Anisoki?

**I: You do not get circumcised?**

R: Eheh.

**R: Yes**

I: Bathi indaba?

**I: Why not?**

R: Eish, bathi uJesu wasoka manje nathi akufanele sisoke, izinto ezinjalo.

**R: They say Jesus was circumcised so there is no need for us to get circumcised, something like that.**

I: UJesu wasoka?

**I: Jesus was circumcised?**

R: Basho njalo?

**R: That is what they say.**

I: Wasoka ngayiphi indlela yena?

**I: How was He circumcised?**

R: Eish, eyi angazi mina.

**R: I do not know.**

I: So, ngalokho-ke ngoba uJesu esokile nina akuvumi ukuthi nina nisoke.

**I: So because Jesus was circumcised you may not be circumcised?**

R: Eheh.

**R: Yes.**

I: Kushiwo njalo?

**I: Is that what they say?**

R: Ehhe, esontweni lethu.

**R: Yes, at our church?**

I: Ehhe.

**I: Yes**

R: Ehhe.

**R: Yes**

I: Uyalazi vese lelo vesi?

**I: Do you know that verse?**

R: Angilazi.

**R: I do not know it.**

I: Kodwa kuye kushiwo njalo?

**I: But that is what they normally say?**

R: Mm, kushiwo njalo.

**R: Mm, that is what they say.**

I: Mm, kuthiwa kwenziwani uma kuthiwa uyasoka wena uma uJesu esokile kwenzakalani?

**I: Mm, what will happen to you if you get circumcised even though Jesus was circumcised?**

R: Abazi kangcono bathi ngeke uwubone umbuso wezulu, angazi.

**R: Those that know better say you will not enter the kingdom of heaven. I do not know.**

I: Ngobani?

**I: Why?**

R: Ngoba wena phela usuphule umthetho.

**R: Because you would have broken the law.**

I: Alright. So, ngalokho-ke wena ene kukubekaphi?

**I: Alright. So, where do you stand because of that?**

R: Mina ngingasoka uma kuthiwa kuyasokwa.

**R: Personally I would get circumcised if I were to be given a chance.**

I: Even though kuphikisa inkolo yakho?

**I: Even though it is against your beliefs?**

R: Yah.

**R: Yeah**

I: Ukuthi phela njengamanje awusokile.

**I: The thing is now you are uncircumcised.**

R: Angisokanga.

**R: I am uncircumcised.**

I: Ehhe, so, into eyenza ukuthi usoke iziphi izinto-ke, ngicela ungibalele nje noma eziwu, ezikuvimbile?

**I: Yes, so what is stopping you from getting circumcised? Please list the things that are preventing you from getting circumcised?**

R: Azikho ezingivimbile, ukuthi nje ngiyasaba nje kuphela.

**R: Nothing is preventing me. It is just that I am scared. That is all.**

I: Usaba ini mhlasimpe, ukuthi kuzokwenzakalani?

**I: What are you afraid of?**

R: uma bengi [Inaudible: 09:08:08] kabi kungenzeka ngife.

**R: If they [inaudible] badly I might die.**

I: Ehhe

**I: Yes.**

R: Ileyonto.

**R: That is the reason.**

I: Uzobe ubulawa yini uma ucabanga, yini engaba engenzeka e-wrong?

**I: What do you think will kill you? What do you think could go wrong?**

R: Mhlampe makwenzeka ngithole udokotela lo osafunda afundele kimi ngoba mina ngizobe ngingamazi phela.

**R: Maybe if I get a trainee doctor who will practice on me, because I will not be aware.**

I: Mm.

**I: Mm**

R: Yah.

**R: Yeah**

I: Manje umbona kanjani ofundile nongafundanga?

**I: So how do you tell the difference between a qualified and a non-qualified doctor?**

R: Kuzobonakala phela uma sengithungeke kabi ukuthi hhayi lona ngati ubefundela kimi.

**R: It will become clear after he has stitched me up badly that the doctor was using me to practice.**

I: Ooh so, njengamanje kodwa uyasaba ngoba ngoba phela awazi noma uzothola lo ofundile noma ongafundanga ngoba ababhaliwe ebusweni.

**I: Oh, so right now you are scared because you do not know whether you will be assisted by a qualified doctor or not because you cannot tell just by looking at them.**

R: Ehhe.

**R: Yes.**

I: Ngizama ukuchaza lokho. alright ngiya understand-ke. So, thina-ke as abantu bezempilo singenzani ukuthi sikukhuthaze wena ukuthi usoke, singakukhombisa kanjani ukuthi kuyinto e-safe?

**I: I am trying to understand that. Alright I understand. So what can we as healthcare practitioners do to motivate you to undergo circumcision? How can we show you that it is a safe procedure?**

R: Angazi.

**R: I do not know.**

I: Igenge le ohlala nayo isokile yona? Like anbangani bakho?

**I: Are your peers circumcised, like your friends?**

R: Abanye basokile, abanye abasokanga.

**R: Some are circumcised. Some are not circumcised.**

I: Ok, kulaba abaosikle bathini bona ngakho?

**I: Ok. What do your circumcised peer say about it?**

R: Bathi ku-right kona ukusoka.

**R: They say being circumcised is good.**

I: Mm, abasitsheli wena njengoba usasaba and even though besho bona?

**I: Mm, do they not tell you since you are scared even though they say that it is good?**

R: Ngiyasaba mina.

**R: I am scared.**

I: Mm. ngqaphandle kukadokotela lo omsabayo ukuthi ene angakwenza kabi, kukhona mhlasimpe enye into ekuvimbe sonke lesikhathi, yona lenkolelo obukhuluma ngayo eka Jes?

**I: Mm, besides the doctor that you are scared of that he might not circumcise you correctly is there perhaps any other factor that has inhibited you all this time, perhaps the belief that you spoke about regarding Jesus?**

R: Hhayi.

**R: No.**

I: Ayikuvimbi yona?

**I: It does not inhibit you?**

R: Hhayi.

**R: No.**

I: But ikona ukuthi inkolo yakho iyasho ukuthi ene umuntu akufanele asoke?

**I: But your beliefs say that people should not circumcise?**

R: Ehhe.

**R: Yes**

I: Kukhona okunye ofuna ukungitshela kona mayelana nakho ukusoka?

**I: Is there anything else that you would like to tell me about circumcision?**

R: Eheh.

**R: No.**

I: La esinga-improve-a khona-ke, like la singenza kanjani nje ukuthi sinigqugquzelele? Ekhaya ukhona osokile yini ekhaya?

**I: Is there perhaps an area that we can improve in? What can we do to motivate you? Is there anyone that is circumcised at home?**

R: Akekekho osokile.

**R: There is no one that is circumcised.**

I: Indaba uma ucabanga engekho ekhaya?

**I: Why do you think that is the case?**

R: Eish, angazi.

**R: I do not know.**

I: Ok, hhayi siyabonga kakhulu bhuti ngosizo lwakho, le information osinikeze yona sicabanga ukuthi izosisiza, yah.

**I: Ok. Thank you very much for your help. We think the information that you gave us will help us a lot.**

END OF TRANSCRIPT
